# Paraganglioma-like dermal melanocytic tumor: a case report

**DOI:** 10.1186/1757-1626-1-48

**Published:** 2008-07-18

**Authors:** Deba P Sarma, Bryan Teruya, Bo Wang

**Affiliations:** 1 Department of Pathology, Creighton University Medical Center, Omaha, NE, 68131, USA

## Abstract

Paraganglioma-like dermal melanocytic tumor is a rare subtype of benign dermal melanocytic tumors. Its histopathologic features resemble those of paraganglioma, but the immunostaining characteristics are those of melanocytic lesions.

We report a case of a 60-year-old male with a paraganglioma-like dermal melanocytic tumor of his left cheek and briefly review the English literature.

## Case presentation

**Figure 1:**
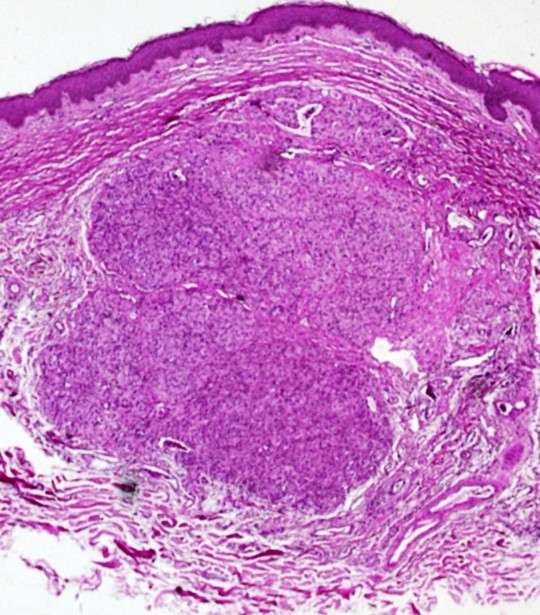


**Figure 2:**
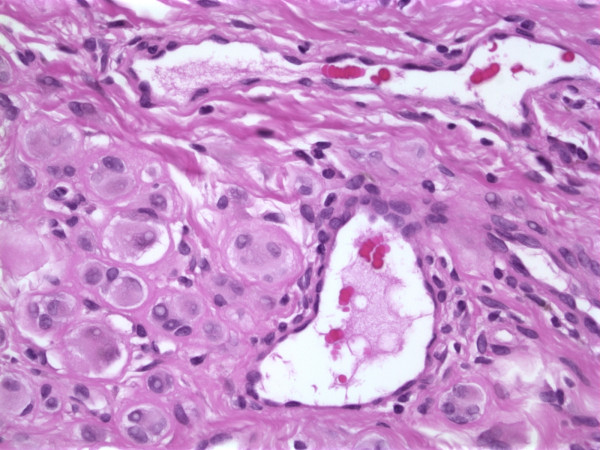


A 60-year-old male presented with a skin-colored slightly raised soft 0.8 cm papule on his left cheek present for an unknown period of time. The clinical impression was a "skin tag". The patient's past medical history was unremarkable. An excisional biopsy of the lesion showed a well demarcated cellular nodule in the dermis (Fig. 1) with normal overlying epidermis. The neoplasm was composed of organoid and nested groups of large epithelioid cells separated by delicate fibrous strands and prominent blood vessels. The epithelioid cells had distinct large nuclei with prominent red nucleoli and somewhat clear cytoplasm (Fig. 2). Some of the large epitheliod cells had multiple nuclei and vacuoles. There was no cytoplasmic melanin pigment or eosinophilic granule. Neither mitotic activity nor necrosis was seen. There was one area of the tumor showing nests of mature nevus cells.

**Figure 3:**
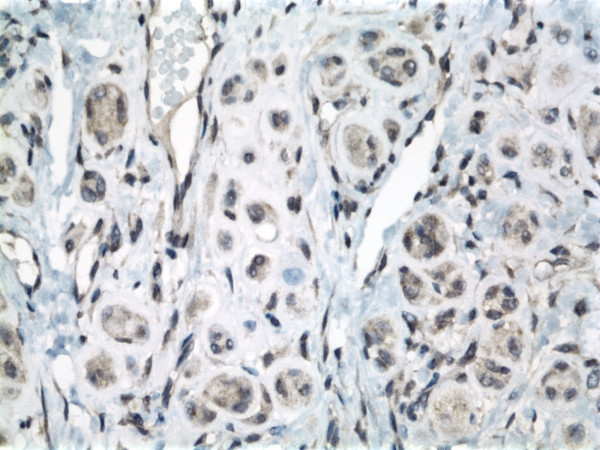


The neoplasm was immunoreactive for Melan-A, MITF, and S-100 protein (Fig. 3) indicating melanocytic lineage of the tumor cells. The tumor cells were negative for CD31, CD34, CD68, cytokeratin AE 1/3 and HMB-45.

The excisional biopsy margin was clear. Clinical follow-up was planned.

## Discussion

The term of primary paraganglioma-like dermal melanocytic tumor (PDMT) was coined by Deyrup, et al [1] in 2004. PDMT is considered as a unique benign neoplasm derived from melanocytes. The lesion may be confused with other benign dermal tumors, such as cellular blue nevus and granular cell tumor or malignant dermal tumors, such as melanoma [2]. The total number of cases described in the English literature is about 8 [1]. It is often seen in patients aged 18–53 with a female preponderance. It is not associated with Carney's syndrome or prior melanoma. Clinically, it presents as a non-pigmented skin nodule averaging 1.4 cm in diameter. Microscopically, the tumor is typically a well demarcated dermal neoplasm with normal overlying epidermis. Junctional melanocytic proliferation or nevoid nest is usually not present. It is comprised of large epitheliod cells in an organoid or nest-like pattern separated by delicate fibrous strands and blood vessels. There is no necrosis but increased mitotic activity can be rarely encountered. These histopathologic features are reminiscent of those of paraganglioma. However, primary cutaneous paraganglioma remains a very rare tumor. Only one such case has been reported in 2006 in the scalp of a 10-year-old boy [3]. The fact that the skin contains nerves and melanocytes but is devoid of ganglia may explain the rarity of cutaneous paraganglioma. The tumor cells of paraganglioma are usually negative for melanocytic markers, such as Melan-A, HMB-45, and MITF. PDMT is considered a variant of benign dermal melanocytic nevus with benign clinical behavior [1]. Other malignant and potentially malignant dermal tumors, such as melanoma and dermal melanocytic tumor of uncertain potential [4] can be excluded because of the absence of any atypical features, such as nuclear atypia, macronucleoli, increased mitotic activity, and necrosis.

The purpose of this report is to familiarize clinicians and pathologists with such a rare type of benign dermal melanocytic tumor.

## Consent

Written consent was obtained from the patient for publication of this case report. A copy of the written consent is available for review by the Editor-in-Chief of this journal.

## Competing interests

The authors declare that they have no competing interests.

## Authors' contributions

DPS conceived, drafted and submitted the manuscript. BW reviewed the literature and prepared the photomicrographs. BT revised and proof-read the manuscript. All authors have read and approved the final manuscript.
